# Concordance for curve type in idiopathic scoliosis among family members

**DOI:** 10.1590/1413-785220172503172684

**Published:** 2017

**Authors:** Clarissa Miranda Carneiro de Albuquerque olbertz, Jérôme Sales de Gauzy, Paulo Cezar Vidal Carneiro de Albuquerque, Frank Accadbled, Paula Eduarda Miranda Carneiro de Albuquerque, José Lamartine de Andrade Aguiar

**Affiliations:** 1 Universidade Federal de Pernambuco, Hospital das Clínicas, Surgery of Department, Recife, PE, Brazil; 2 Centre Hospitalier Universitaire de Toulouse, Hôpital des Enfants, Department of Pediatric Orthopedic Surgery, Toulouse, France; 3 Faculdade Pernambucana de Saúde, Instituto de Medicina Integral Professor Fernando Figueira, Recife, PE, Brazil

**Keywords:** Scoliosis, Genetics, Spine

## Abstract

**OBJECTIVE:**

To evaluate the concordance for the curve pattern, side and levels of the superior apical vertebrae, apex and inferior apical vertebrae of curves in patients and their relatives with idiopathic scoliosis.

**METHODS:**

Concordance according to the Lenke classification for curve pattern, side and levels of the superior apical vertebrae, apex and inferior apical vertebrae were evaluated comparative and prospectively in 243 pairs of patients and respective relatives with idiopathic scoliosis.

**RESULTS:**

The family concordance for the curve pattern and side was 51.4% (125 pairs). Among these pairs, the concordance of the levels of the vertebrae was 91.2% (114 pairs). The concordance rate for the curve pattern and side between parents/children was 51.6% and between siblings was 50.0% (p-value= 0.411). The concordance rates of the levels of vertebrae were 86.8% and 95.1%, respectively (p-value = 0.219).

**CONCLUSION:**

Curve shape in idiopathic scoliosis is related to family and degree of kinship, since the data showed a high concordance for the curve pattern, side and levels of the apical vertebrae and apex between patients and relatives with this deformity. The concordance was higher in those with a closer degree of kinship.***Level of Evidence II, Lesser Quality Prospective Study.***

## RESUMO

**OBJETIVO:**

Avaliar a concordância para o padrão de curva, lado e *níveis das vértebras apical superior, ápex* e apical inferior das curvas de pacientes e respectivos familiares com escoliose idiopática.

**MÉTODOS:**

A concordância, pela classificação de Lenke, para o padrão de curva, lado e níveis das vértebras apical superior, ápex e apical inferior foi avaliada em 243 pares de pacientes e respectivos familiares com escoliose idiopática.

**RESULTADOS:**

A concordância familiar para o padrão de curva e lado foi de 51,4% (125 pares). Entre esses pares, a concordância dos níveis das vértebras foi de 91,2% (114 pares). A taxa de concordância para o padrão de curva e lado entre pais/filhos foi de 51,6% e entre irmãos foi de 50,0% (p= 0,411). As taxas de concordância dos níveis das vértebras foram respectivamente de 86,8% e 95,1% (p-valor = 0,219).

**CONCLUSÃO:**

O formato das curvas na escoliose idiopática tem relação familiar e com o grau de parentesco, uma vez que se reportou alta concordância para o padrão de curva, lado e níveis das vértebras apicais e ápex entre pacientes e familiares com a deformidade. A concordância foi maior entre aqueles com grau de parentesco mais próximo. Nível de Evidência Ii, Estudo Prospectivo de Menor Qualidade.

## INTRODUCTION

Idiopathic scoliosis (IS) is a three-dimensional deformity of the spine in which a structural lateral curvature is associated with vertebral rotation and lordosis.^1^ This deformity affects otherwise healthy patients and is one of the most common pathologies involving the spine.^2^

Despite many years of research, the exact cause of this condition has not yet been found. Several hypotheses have included metabolic, biomechanical, neuromuscular, developmental and genetic factors.^2^ IS is often seen in several members of the same family, strongly suggesting a genetic component.^2^ ^-^ ^5^ One study showed that 11% of first-degree relatives of patients with IS are also affected, as well as 2.4% and 1.4% of second and third-degree relatives, respectively.^5^ Studies on twins reported higher concordance for the presence of the curve in monozygotic twins in comparison with dizygotic twins.^6^ ^,^ ^7^

As early as the 1950s scientists suggested that the shape of the curve in IS was genetically determined.^8^ Support for this theory was reported in other studies which identified similar curves in twins concordant for IS.^9^ ^-^ ^11^

Curve pattern has not been widely investigated in familial IS other than in twin pairs. The aim of this study was to evaluate whether patients and respective family relatives with IS have concordant curve types. To do so, we compared the pattern, the side and the levels of the apical superior vertebrae (ASV) and the apex and apical inferior vertebrae (AIV) of curves in patients with IS and their relatives.

## MATERIAL AND METHODS

The data collected followed the institutional review board standards on human experimentation (protocol number 08-0916). A total of 419 individuals with a positive family history for IS were referred to our institution between 2006 and 2015. After excluding 21 subjects who did not have spine X-rays for evaluation, 398 patients remained. The present study used only data contained in patient charts and the researchers signed an agreement to update this data.

IS was prospectively assessed using full spine standing posteroanterior X-rays analyzed by 2 observers. For diagnosis, this study considered lateral structural curvature greater than 10° according to Cobb.^12^

Concordance according to the Lenke classification for curve pattern and the side of the convexity of the curves were analyzed.^13^ In this study the double major (DM) curves were not differentiated from the thoracolumbar/lumbar-main thoracic curves (TL/L-MT) and 5 different patterns were identified. The ASV, apex and AIV of each curve were subsequently identified.^14^

We compared the X-rays in pairs containing the patient and their respective family relative with IS. A total of 21 families presented more than 2 family relatives with IS and all were considered in the study. These pairs were identified with respect to type of family relationship and individual sex. First-degree family relationships were parents, siblings and children; second-degree relationships were uncles/aunts, nephews/nieces, grandparents/grandchildren; third-degree relationships were cousins.^15^ A total of 243 pairs were evaluated: 225 first-degree relatives, 6 second-degree relatives and 12 third-degree relatives. The first-degree pairs were comprised of 159 siblings and 66 parents/children. The second-degree pairs contained 4 uncles/aunts/nephews/nieces and 2 grandparents/grandchildren. The third-degree pairs were comprised of 12 cousin pairs. In total, there were 174 pairs of female patients, 14 male pairs and 55 pairs with one male and one female subject. The sibling pairs were comprised of 110 female pairs, 11 male pairs and 38 female/male pairs. The parent/child pairs were comprised of 52 female pairs, 1 male pair and 13 female/male pairs.

**Figure 1:**
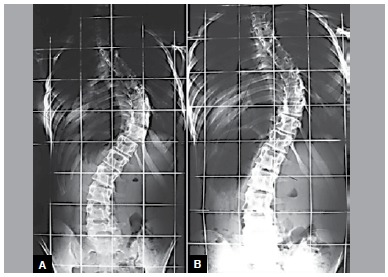
Radiographs of two twin sisters with concordant curve pattern, side and levels of ASV, apex and AIV. A, DM Curve; right/left; ASV T5, apex 9 and AIV T12/ASV L1, apex L4, AIV L5. B, DM Curve; right/left; ASV T5, apex T9, AIV T12/ASV L1, apex L4, AIV L5.

The pairs were considered concordant if both relatives had the same curve pattern and side. They were compared with respect to the level of ASV, apex and AIV. The study considered individuals to have the same curve type when there was no difference in the position of these vertebrae in a maximum of two levels, proximal or distal. (Figure 1)

A Microsoft Excel spreadsheet using EPI INFO version 3.5.2 was used to analyze the data. To evaluate the chance of concordance of the curve pattern, side and levels of the vertebrae, the prevalence was calculated in the different pairs according to family relationship. The chance of concordance was then compared using the chi-square test. The same aspects were also verified according to sex: female/female (FxF); male/male (MxM); female/male (FxM). All conclusions were obtained using a 5% significance level.

## RESULTS

The study evaluated 243 pairs of individuals, 125 (51.4%) of which were concordant for the curve pattern and side of the deformity. Among these, there was a high concordance prevalence for the levels of ASV, apex and AIV: 91.2% (114 pairs).

Distribution of the curve patterns according to family relationship is shown in Table 1. Higher concordance prevalence was seen in all groups for DM or TL/L-MT curves. The proportion comparison test was significant in all evaluated groups (all with p-value <0.05). (Table 1)

**Table 1:** Concordance prevalence of the curve pattern according to family relationship.

Curve pattern	ALL		Parent/child		Siblings		Other^2^	
	Total	Concordance	Total	Concordance	Total	Concordance	Total	Concordance
MT	31	0 (0.0%)	6	0 (0.0%)	21	0 (0.0%)	4	0 (0.0%)
DT	15	2 (13.3%)	3	0 (0.0%)	11	2 (18.2%)	1	0 (0.0%)
DM or TL/L-MT	283	184 (65.0%)^1^	93	68 (73.1%)^1^	169	106 (62.7%)^1^	21	10 (47.6%)^1^
TL/L	87	26 (29.9%)	19	6 (31.6%)	60	20 (33.3%)	8	0 (0.0%)
TM	70	38 (54.3%)	11	2 (18.2%)	57	36 (63.2%)	2	0 (0.0%)

^1^Greater concordance for DM or TL/L-MT curves in all groups (p-valor < 0.05). ^2^Other refers to second - and third-degree relatives.

Table 2 shows the distribution of the evaluated pairs in relation to type of family relationship and concordance prevalence for each evaluated pair type. The concordance prevalence for the curve pattern and side was of 57.6% for parents/children, 51.6% for siblings and 8.3% for cousins. The proportion comparison test was not significant between the groups of siblings and parents/children (p= 0.411). However, when comparing the chance in the cousin group with the sibling and the parent/child groups, the test was significant (p= 0.004 and 0.002, respectively). The concordance evaluation of vertebrae was of 95.1% for siblings and 86.8% for parents/children. The proportion comparison test was not significant (p= 0.219).

**Table 2:** Concordance prevalence of curve pattern, side and vertebrae according to family group.

Family relationship	Total	Concordance evaluated	
		Curve and side	ASV, Apex, AIV
Siblings	159 (65.4%)	82 (51.6%)^1^	78 (95.1%)^3^
Parent/Child	66 (27.2%)	38 (57.6%)^1^¹	33 (86.8%)^3^
Aunt-Uncle/Niece-Nephew	4 (1.6%)	2 (50.0%)	2 (100.0%)
Grandparent/Grandchild	2 (0.8%)	2 (100.0%)	1 (50.0%)
Cousin/Cousin	12 (4.9%)	1 (8.3%)^2^	0 (0.0%)
Total	243 (100.0%)	125 (51.4%)	114 (91.2%)

^¹^Similar prevalence between Siblings and Parent/Child (p= 0.411). ^²^Prevalence significantly lower among Cousins, compared to Siblings and Parent/Child groups (p= 0.004 and 0.002, respectively). ^³^Prevalence statistically similar between Siblings and Parent/Child (p= 0.219).

Table 3 shows the distribution of the evaluated pairs in relation to sex and the concordance prevalence of the curb pattern, side and levels of the vertebrae. The concordance prevalence of the curve pattern and side was highest for the FxF comparison (55.8%), followed by FxM (45.6%) and MxM (21.4%). The proportion comparison test was significant for the differences found (p= 0.028). The concordance comparison found for vertebrae was of 100% among MxM pairs, followed by FxF pairs (91.7%) and FxM pairs (88.5%). The proportion comparison test was not significant (p= 0.904).

**Table 3:** Concordance prevalence of curve pattern, side and vertebrae according to sex.

Sex	Total	Concordance evaluated	
		Curve and side	ASV, Apex, AIV
FxF	172 (70.8%)	96 (55.8%)^1^	88 (91.7%)^22^
MxM	14 (5.8%)	3 (21.4%)	3 (100.0%)^22^
FxM	57 (23.5%)	26 (45.6%)^1^	23 (88.5%)^2^
Total	243 (100.0%)	125 (51.4%)	114 (91.2%)

^¹^Similar prevalence between FxF and FxM (p= 0.028). ²Similar prevalence between FxF, MxM and FxM (p= 0.904).

Table 4 shows the concordance prevalence for the curve pattern, side and vertebrae according to sex in the sibling and parent/child groups. In the sibling group the highest prevalence of concordance for the curve pattern and side was in the FxF pairs (59.1%), followed by FxM (36.8%) and MxM (27.3%). The proportion comparison test showed a significant difference in the percentages found (p= 0.015). In comparing vertebrae concordance, 100% of these concordant cases for MxM and FxM comparisons were also concordant. In the FxF pairs this percentage was 93.8%. Since two groups presented total concordance, the application of the proportion comparison test was not feasible. For the parent/child group, the highest prevalence of concordance for the curve pattern and side was seen in the FxM pairs (69.2%), followed by FxF (55.8%). The proportion comparison test was not significant (p= 0.378). Of the FxF pairs concordant to the curve pattern and side, 93.1% were concordant for the vertebrae. In the FxM pairs, this percentage was 66.7%. The proportion comparison test was not significant (p= 0.137).

**Table 4:** Concordance prevalence of curve pattern, side and vertebrae according to gender in the "siblings" and "parents/child" groups.

Sex	Siblings			Parent/child		
	Total	Concordance evaluated		Total	Concordance evaluated	
		Curve and side	AIS, Apex, AIV		Curve and side	AIS, Apex, AIV
FxF	110 (69.2%)	65 (59.1%)^1^	61 (93.8%)^2^	52 (78.8%)	29 (55.8%)^3^	27 (93.1%)^4^
MxM	11 (6.9%)	3 (27.3%)	3 (100.0%)^2^	1 (1.5%)	0 (0.0%)	-
FxM	38 (23.9%)	14 (36.8%)	14 (100.0%)^2^	13 (19.7%)	9 (69.2%)^3^	6 (66.7%)^4^
Total	159 (100.0%)	82 (51.6%)	78 (95.1%)	66 (100.0%)	38 (57.6%)	33 (86.8%)

^¹^Higher prevalence in FxF (p= 0.015). ^²^Similar prevalence between FxF, MxM and FxM. ^3^Similar prevalence between FxF and FxM (p= 0.378). ^4^Similar prevalence between FxF and FxM (p= 0.137).

Of the total of 21 families, 19 allowed combination of 3 pairs of individuals with IS; in 2 other families, 6 pairs were made. In 3 families (14.3%), all pairs evaluated for curve pattern and side were concordant. In 5 families (23.8%), all were discordant. The study identified 16 families (76.2%) with one or more pairs which were concordant for the curve pattern and side. Vertebrae assessment found that in 15 (93.8%) of these 16 families there were one or more concordant pairs. (Table 5)

**Table 5:** Number of concordant and discordant pairs in evaluating curve pattern, side and vertebrae in families.

Evaluated family	TOTAL pairs	Curve and side		Vertebrae	
		Concordant pairs	Discordant pairs	Concordant pairs	Discordant pairs
1	3	1	2	1	-
2	3	1	2	-	1
3	3	-	3	-	-
4	3	-	3	-	-
5	3	1	2	1	-
6	3	1	2	1	-
7	3	1	2	1	-
8	3	1	2	1	-
9	3	1	2	1	-
10	3	-	3	-	-
11	3	-	3	-	-
12	3	1	2	1	-
13	3	3	-	3	-
14	6	3	3	3	-
15	3	1	2	1	-
16	3	1	2	1	-
17	3	3	-	3	-
18	3	-	3	-	-
19	6	3	3	3	-
20	3	3	-	3	-
21	3	1	2	1	-

## DISCUSSION

IS is a genetic disorder involving one or more genetic loci and complex interactions between them for expression.^3^ Several studies have concluded that the most likely form of inheritance is multifactorial, postulating that predisposing alleles are required together with environmental factors to express this phenotype.^2^ ^,^ ^9^ ^,^ ^15^

Studies have suggested a biomechanical explanation for family tendency in IS when proposing that it originates in a genetically determined spine profile. Individuals with flatter profiles would be more vulnerable to developing the deformity.^1^ ^,^ ^7^ ^,^ ^16^ Other studies argue that the primary mechanical factor triggering this disorder is rotational instability. Subsequent shear forces act on certain areas of the spine and lead to rotation of the vertebral bodies, producing apical lordosis and the appearance of lateral deviation in the anteroposterior plane in X-rays.^17^ ^,^ ^18^

In the 1950s Ponseti and Friedman^8^ suggested that the shape of the curve in IS was genetically determined and other studies have provided support for this theory.^3^ ^,^ ^4^ ^,^ ^7^ ^,^ ^8^ ^,^ ^10^ ^,^ ^19^ Dryden et al.^20^ observed that similar back shapes were more frequently found in individuals who were closer genetically and of the same sex, suggesting a relationship between deformity and sex. Kesling and Reinker^9^ demonstrated that monozygotic twins tend to have more similar curves than dizygotic twins and only one segment difference in apex vertebrae position. Van Rhijn et al.^10^ reported that the direction of the convexity of the curve and apex vertebrae were most commonly the same in monozygotic twins with scoliosis. 

Our results demonstrate that the concordance for curve pattern and side among family relatives with IS is high. In an analysis of 100 families Sales de Gauzy et al.^3^ showed a 66% concordance rate for curve pattern and side in family relatives with IS, with a concordance rate that was not statistically different between siblings (65%) and parents/children (67%). 

In the present study 243 pairs of relatives were evaluated, showing a concordance rate of 51.4% for curve pattern and side, as well as a statistically insignificant (p= 0.411) concordance rate between parents/children (57.6%) and siblings (51.6%), indicating that the chance of concordance between siblings is similar to the rate in parents/children. Comparison between cousins showed a concordance rate of 8.3%, significantly lower than in siblings and parents/children (p= 0.004 and 0.002, respectively), indicating that the chance of concordance between cousins is significantly lower than in these groups. This suggests that genetically more distant family relatives are less likely to present concordance for curve pattern and side.

In this analysis, 114 of the 125 (91.2%) pairs concordant for curve pattern and side were concordant for levels of ASV, apex and AIV. When comparing the different family groups, siblings (95.1%) and parents/children (86.8%) showed no statistically significant difference (p= 0.219), demonstrating that the chance of concordance in vertebra position is similar between these two groups. Because the samples of second - and third-degree relatives were so small, a larger population should be evaluated to accurately compare these groups. We found no studies in the literature comparing vertebra position in family pairs. One study which only evaluated the level of the apex vertebrae in 68 pairs of twin siblings with IS observed that the apex vertebra only differed by one segment in most of the monozygotic pairs.^9^ Another analysis in 18 twin pairs observed that nine-tenths of the apex vertebrae were the same or differed only by one segment between pairs.^10^

In analyzing sex, Sales de Gauzy et al.^3^ found a concordance rate of 68% for curve pattern and side in FxF pairs and 62% in FxM pairs, with no statistically significant difference. 

This analysis showed that the concordance rate for the curve pattern and side was significantly higher (p= 0.028) in FxF (55.8%) and FxM pairs (45.6%) compared with MxM pairs (21.4%), indicating a higher probability of concordance in FxF and FxM pairs than in MxM pairs. However, when vertebra levels were compared among these pair groups, the concordance was not statistically different (p= 0.904), showing that although the concordance for curve pattern and side is greater when comparing FxF and FxM pairs, vertebra position presents similar concordance among all three parameters. In the evaluating curve pattern and side in parents/children, the concordance prevalence was greater in FxM (69.2%) and FxF (55.8%) pairs, again with no statistically significant difference (p= 0.378). In the sibling group, prevalence was significantly higher in FxF pairs (p= 0.015). The analysis of the vertebrae in these groups also showed similar concordance among all pairings.

Both the DM and TL/L-MT curves have structured thoracic and thoracolumbar/lumbar curves but differ in the main curve.^13^ This study opted not to differentiate these curved because it is possible that a structured curve initially identified as minor might intensify in a future assessment. Since the objective of this study was to assess whether pairs had the same curve types, we believe that differentiating DM from TL/L-MT patterns could lead to a false interpretation that similar curves are different, thereby excluding them from the second stage of the study which compared vertebra position. In the analysis by Sales de Gauzy et al.,^3^ the curved patterns most commonly found were DM, followed by TL/L, MT, TL/L-MT and DT. In this study, DM or TL/L-MT patterns were significantly the most frequent in all analyzed groups (p <0.05), followed by TL/L, TM, MT and DT patterns.

In only 14.3% of the 21 assessed families were all pairs concordant. Most families (76.2%) presented one or more concordant pairs and consequently the concordance for curve pattern and side did not increase when more members of the same family were affected by the deformity.

## CONCLUSION

The analysis in this study showed high concordance in curve pattern and side among family relatives with IS and also showed that genetically more distant individuals have less chance of concordance than closer relatives. All evaluated pairs showed a greater frequency of concordance when at least one individual in the pair was female. When evaluating families with the deformity, the findings indicate that the pairs of individuals with IS within a single family are independent of each other. These results reinforce the hypothesis that the type of the curve is related to family and sex and opens prospects for future research including evaluating the concordance among the type of curve and the degree of family relatedness and sex.
